# Prenatal inflammation exposure accelerates lung cancer tumorigenesis in offspring mouse: possible links to IRE1α/XBP1-mediated M2-like polarization of TAMs and PD-L1 up-expression

**DOI:** 10.1007/s00262-024-03666-w

**Published:** 2024-03-30

**Authors:** Jingbo Ma, Jian Tan, Weiqiang Zhang, Miaochun Bai, Keqiang Liu

**Affiliations:** Department of Thoracic Surgery, Seventh Medical Center of Chinese, PLA General Hospital, No. 5, Nanmencang, Dongcheng District, Beijing, 100010 China

**Keywords:** Prenatal inflammation exposure, Lung cancer, Endoplasmic reticulum stress, Tumor-associated macrophage polarization, PD-L1

## Abstract

**Background:**

Prenatal inflammation exposure (PIE) can increase the disease susceptibility in offspring such as lung cancer. Our purpose was to investigate the mechanisms of PIE on lung cancer.

**Methods:**

Prenatal BALB/c mice were exposed to lipopolysaccharide (LPS), and then, their offspring were intraperitoneally instilled with urethane to establish the two-stage lung cancer carcinogenesis model. At the 48 weeks of age, the offspring mice were killed and lung tissues were collected for HE, immunohistochemistry, immunofluorescence, and Luminex MAGPIX®-based assays. CD11b + F4/80 + tumor-associated macrophages (TAMs) were sorted out from lung tumor tissues by cell sorting technique. Flow cytometry was employed to evaluate the extent of M2-like polarization of TAMs and PD-L1 expression.

**Results:**

The offspring of PIE mice revealed more lung lesion changes, including atypical hyperplasia and intrapulmonary metastases. The number of lung nodules, lung organ index, and PCNA, MMP-9 and Vimentin positive cells in lung tissue of PIE group were higher than those of Control group. The increases of mRNA encoding M2 macrophage markers and cytokines in offspring of prenatal LPS-treated mice confirmed the induced effect of PIE on macrophage polarization. Additionally, PIE treatment increased the percentage of CD163 + CD206 + cells in the sorted TAMs. Importantly, endoplasmic reticulum (ER) stress-markers like GRP78/BIP and CHOP, p-IRE1α and XBP1s, and PD-L1 were up-regulated in TAMs from PIE group. Besides, we also observed that IRE1α inhibitor (KIRA6) reversed the M2-like TAMs polarization and metastasis induced by PIE.

**Conclusions:**

IRE1α/XBP1-mediated M2-like TAMs polarization releases the pro-tumorigenic cytokines and PD-L1 expression, which may be the regulatory mechanism of accelerating lung cancer in offspring of mice undergoing PIE.

**Supplementary Information:**

The online version contains supplementary material available at 10.1007/s00262-024-03666-w.

## Introduction

Lung cancer, as the leading public health problems, can cause approximately 350 deaths each day [1], which is characterized by high proliferation rate, significant tendency to early metastasis and poor prognosis [2]. Exposure to tobacco and radiation, presence of chronic lung diseases, and family history are the known risk factors for lung cancer [3]. Recently, prenatal inflammation exposure (PIE) has been shown to accelerate tumorigenesis including lung cancer [4, 5]. PIE, as a key event during pregnancy, can be caused by bacterial, viral, and microbial infections as well as chronic inflammatory states such as prenatal hypertension, diabetes, and smoking, especially in the past years due to the presence of COVID-19 outbreaks [6]. These evidences suggest that PIE may be a risk factor for increased susceptibility to lung cancer in offspring, but the specific mechanisms by which PIE influences on the growth and metastasis of lung cancer in offspring need to be further investigated.

Tumor-associated macrophages (TAMs) are one of the key molecules connecting inflammation and cancer [7]. In general, macrophages mainly include M1 type and M2 type macrophages. To realize their multiple functions, macrophages are polarized toward M1, which contribute to antitumor responses, or M2, which are relevant with tumor progression [8]. TAMs, mostly have the M2-like phenotype and functions, strongly impact the cancer progression via immunosuppressive activities [9]. In lung cancer, prior study has verified that TAMs can stimulate transcription factors, thereby promoting the growth and metastasis of lung cancer [10]. Additionally, Liu et al. [11] have uncovered that lipopolysaccharide (LPS)-induced chronic inflammation increases the susceptibility of lung cancer under exposure to tobacco carcinogen. However, it is unclear whether exposure to LPS-induced chronic inflammation during pregnancy affect the growth and metastasis of lung cancer in offspring through TAMs.

Accumulating evidence has demonstrated the roles of endoplasmic reticulum (ER) stress on the regulation of TAMs function [12]. Endoribonuclease/protein kinase IRE1-like protein (IRE1α)/X-box binding protein 1 (XBP1) is the most commonly branch of unfolded protein response (UPR), and activation of these signaling cascades leads to impaired ER homeostasis [13, 14]. Additionally, XBP1 activation facilitates pro-tumor function of TAMs, thus accelerating the growth and metastasis of colon cancer, which has been verified in prior study [15]. Moreover, programmed death-ligand 1 (PD-L1), an immune checkpoint, has been stated to be influenced by IRE1α signaling in tumor-infiltrating macrophages [16]. Accordingly, we hypothesized the mechanism by which PIE accelerates lung cancer tumorigenesis in offspring mice, possible links to IRE1α/XBP1-mediated M2-like TAMs polarization and PD-L1 up-expression.

## Materials and methods

### Experimental animals

Male and female BALB/c mice (6-week-old) were purchased from Hunan Slake Jingda Laboratory Animal Co., Ltd (Changsha, Hunan). All experiments were conducted with the approval of Animal Care and Use Committee (VS212601464).

### Experimental design

**Fig. 1:**
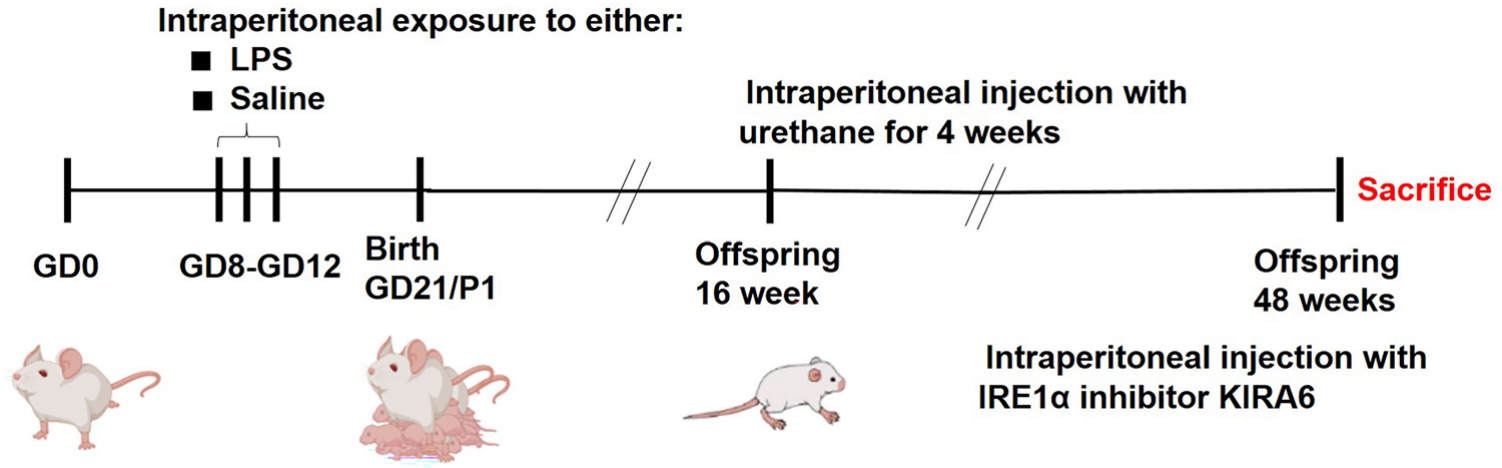
The flow diagram of experimental design

Male/female (1:2) mice were placed in the same cage, and pregnancy was judged by the occurrence of vaginal plug (gestation day, GD0). F0 pregnant mice were intraperitoneally administrated with lipopolysaccharide (LPS, 50 μg/kg, SMB00610, Sigma-Aldrich, St. Louis, MO, USA) to stimulate PIE model at gestation days 8, 10, and 12; F0 pregnant mice in the Control group were injected with equal volume of saline. No deaths or miscarriage occurred in F0 pregnant mice after LPS injection. At 16 weeks of age, the offspring mice were intraperitoneally instilled with urethane (1 g/kg, weekly, U2500, Sigma-Aldrich, St. Louis, MO, USA) to induce lung cancer for 4 weeks [5]. To detect the roles of IRE1α, IRE1α inhibitor (KIRA6, HY-19708, 10 mg/kg, MCE, Shanghai, China) was intraperitoneally injected once per week after induction of lung cancer model. At the 48 weeks of age, mice were killed and lung tissue was collected for the further experiments. The flow diagram of experiment design is expressed in Fig. 1.

### HE staining

Lung tumor tissues were fixed with 4% paraformaldehyde, embedded, and cut into 4-µm slides. Subsequently, the slices were dewaxed, rehydrated, and stained with hematoxylin–eosin (HE). Images were captured by microscope (Ti-s, Nikon, Tokyo, Japan).

### Immunohistochemistry assay

After dewaxing and hydration, the slides were immersed in protease K to antigen repair, exposed to 3% hydrogen peroxide, and incubated with 10% goat serum to block. Following, the slides were incubated with primary antibodies (PCNA, 1:200, sc-56; MMP-9, sc-393859, 1:250, Vimentin, sc-373717, 1:200, Santa Cruz Biotechnology, Inc, Santa Cruz, CA, USA), and then HRP-labeled IgG (SA00001-1, 1:20,000, Proteintech, Wuhan, China) was added. The sections were dyed with 3,3'-diaminobenzidine (DAB) and captured by microscopy (Ti-s, Nikon, Tokyo, Japan). The intensity was measured using ImageJ software (NIH, Bethesda, MD, USA).

### Estimation of cytokine levels

Whole lung tissues were cut into small pieces, lysed using RIPA buffer, and protein concentration was estimated using BCA kit (E-BC-K318-M, Elabscience, Wuhan, China). Protein levels of cytokine were measured by Luminex MAGPIX®-based assay (Bio-Rad, Hercules, CA, USA) according to manufacturer’s protocol.

### TAMs polarization and flow cytometry analysis

Referring to prior study [17], lung tumor tissues from offspring were extracted after 48 weeks of age, and CD11b + F4/80 + TAMs (eFluor450-labeled anti-CD11b, 48–0112-82; AlexaFluor 488-labeled anti-F4/80, 53-4801-82; eBioscience, San Diego, CA, USA) were sorted out by cell sorting technique, that is, TAMs in lung cancer tissues. To evaluate the PIE on extent of TAMs M2-like polarization, the purified macrophages were stained with FITC-conjugated anti-CD163 (11-1631-82, Invitrogen, Carlsbad, CA, USA) and APC-conjugated anti-CD206 (17–2061-82, Invitrogen, Carlsbad, CA, USA) and detected by flow cytometry (BD Biosciences, Franklin Lakes, New Jersey, USA).

### RT-qPCR assay

Total RNA from lung tumor tissues was extracted with TRIzol Reagent (abs9331, Absin, Shanghai, China). Subsequently, cDNA was synthesized and real-time PCR was proceeded by One-step reverse transcription PCR kit (FP313-01, Tiangen, Beijing, China). β-actin was regarded as internal control for quantification of mRNAs. The primer sequences were listed as following: Adgre1 (F4/80) forward, 5’-GCCACGGGGCTATGGGATGC-3’, reverse, 5’-ACCCACAGTGTCCAGGCAAGG-3’; Arg-1 forward, 5’-CTTGCGAGACGTAGACCCT-3’, reverse, 5’-AATCGGCCTTTTCTTCCTTCC-3’; Mrc1 (CD206) forward, 5’-GGAAACGGGAGAACCATCAC-3’, reverse, 5’-GGCGAGCATCAAGAGTAAAG-3’; iNOS forward, 5’-CCTTGGTGAAGGGACTGAGC-3’, reverse, 5’-CAACGTTCTCCGTTCTCTTGC-3’; CD86 forward, 5’-GACCGTTGTGTGTGTTCTGG-3’, reverse, 5’-GATGAGCAGCATCACAAGGA-3’; VEGFA forward, 5’-TTCATGGATGTCTACCAGCGAA-3’, reverse, 5’-CACTCCAGGGCTTCATCGTT -3’; PD-L1 forward, 5’-TGCGGACTACAAGCGAATCACG-3’, reverse, 5’-CTCAGCTTCTGGATAACCCTCG-3’; β-actin forward, 5’-AGGCCCAGAGCAAGAGAGGTATC-3’, reverse, 5’-CGCAGCTCATTGTAGAAGGTGTG-3’.

### Western blotting

Lung tumor tissues and cells were lysed in RIPA lysis buffer and BCA kit was adopted to test the concentration of total protein (E-BC-K318-M, Elabscience, Wuhan, China). Then, protein was separated, and transferred into PVDF membrane. After blocking with skimmed milk, the membranes were incubated overnight anti-IRE1α (27,528-1-AP, 1:2000), anti-XBP1s (24,868-1-AP, 1:6000), anti-GRP78/BIP (GRP78/BIP, 1:10,000), anti-CHOP (15,204-1-AP, 1:3000), anti-PERK (24,390-1-AP, 1:2000), and anti-β-actin (81,115-1-RR, 1:20,000) (Proteintech, Wuhan, China); anti-E-cadherin (#3195, 1:1000), and anti-N-cadherin (#13,116, 1:1000) (CST, Boston, MA, USA); and Vimentin (sc-373717, 1:5000, Santa Cruz Biotechnology, Inc, Santa Cruz, CA, USA). Second antibody (anti-Rabbit IgG-HRP, SA00001-2, 1:10,000, Proteintech, Wuhan, China) was subsequently incubated for 2 h. Finally, immunoblots were performed by the enhanced chemiluminescence (ECL) kit (34,577, Thermo Fisher Scientific, Waltham, MA, USA), and visualized by Tanon 5200 (Tanon, Shanghai, China).

### Immunofluorescence assay

Paraffin-embedded lung tumor tissues were cut into 4 μM slides, followed by blocking with 10% goat serum and incubating with corresponding primary antibodies: rabbit anti-XBP1s (24,868-1-AP, 1:400, Proteintech, Wuhan, China) and anti-CD206 (1:400, 60,143-1-Ig, Absin, Shanghai, China) overnight. Subsequently, corresponding Alexa Fluor secondary antibodies (abs20025, Absin, Shanghai, China) were added and incubated at room temperature. The sections were dyed with DAPI to display the nuclei location, and captured by microscopy (Ti-s, Nikon, Tokyo, Japan).

### Cell lines and lines and conditioned media

Lung cancer cells (A549 and SPC-A-1) were purchased from Procell (Wuhan, China) or Otwo Biotech (Guangzhou, China), and cultured in Ham's F-12 K (PM150910, Procell, Wuhan, China) or RPMI-1640 medium (PM150110, Procell, Wuhan, China) supplemented with 10% FBS (164,210, Procell, Wuhan, China) and 1% Penicillin–Streptomycin (PB180120, Procell, Wuhan, China).

### Transwell assay

A549 and SPC-A-1 cells in serum-free medium were seeded in upper chamber with Matrigel (354,480, BD Biosciences, Franklin Lakes, New Jersey, USA). The bottom chamber was filled with Ham's F-12 K (PM150910, Procell, Wuhan, China) or RPMI-1640 medium (PM150110, Procell, Wuhan, China) supplemented with 10% FBS (164,210, Procell, Wuhan, China). After incubation for 6 h, the upper chambers were replaced with conditioned medium (CM) generated by TAMs from Control and PIE groups. Cells invasion to the bottom chambers were stained with 0.1% crystal violet and counted on a light microscope (Ti-s, Nikon, Tokyo, Japan).

### Statistical analysis

Data are presented as the mean ± standard deviation (SD) and analyzed using SPSS 22.0 or GraphPad Prism 7. The comparisons between the two groups were made using Student’s *t* test, while multiple groups was analyzed by one-way ANOVA followed by LSD test. *P* < 0.05 was deemed to be statistically significant.

## Results

### PIE drove tumorigenesis of lung cancer in female mice offspring

**Fig. 2:**
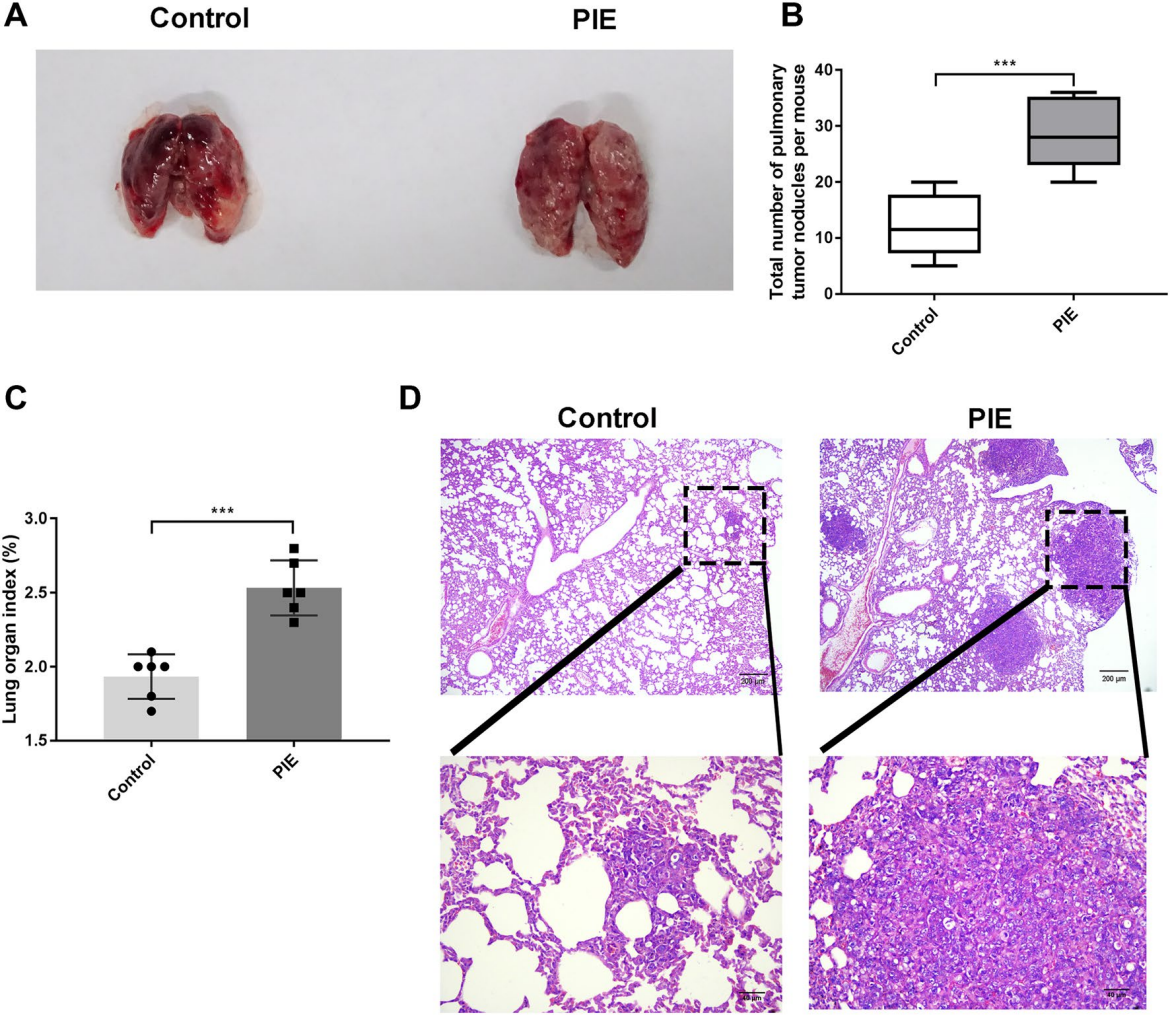
PIE drove tumorigenesis of lung cancer in female mice offspring. **a** The macroscopically images of lung cancer in female mouse offspring at 48 weeks were shown; **b** the number of lung surface tumor nodules in the PIE and Control group was compared; **c** lung organ index of PIE group in the PIE and Control group was compared; **d** the lung lesion changes in the offspring of LPS-treated prenatal mice were detected by HE staining, scale bar = 200 μM or 40 μM; ***P < 0.001; PIE, prenatal inflammation exposure; data were expressed as mean ± standard deviation (SD), *n* = 6 mice in each group

Prior study has clarified prenatal exposures can lead to increases of malignancies in offspring [18]. To investigate the impacts of PIE on lung tumorigenesis, two stage carcinogenesis protocol of lung cancer in BALB/c mice was adopted in this study. Figure 2a presents the macroscopically images of lung cancer in female mice offspring at 48 weeks. The number of lung surface tumor nodules in PIE group was higher than that of Control group (Fig. 2b). Additionally, lung organ index of PIE group was obviously increased at 48 weeks of age by comparison with Control group (Fig. 2c). HE analysis revealed more lung lesion changes, including intrapulmonary metastasis in the offspring of LPS-treated prenatal mice (Fig. 2d). These evidences suggested that PIE may be a predisposing factor for stimulating lung cancer in offspring.

### PIE-enhanced cell proliferation and epithelial-mesenchymal transition (EMT) in female offspring mice with lung cancer

**Fig. 3:**
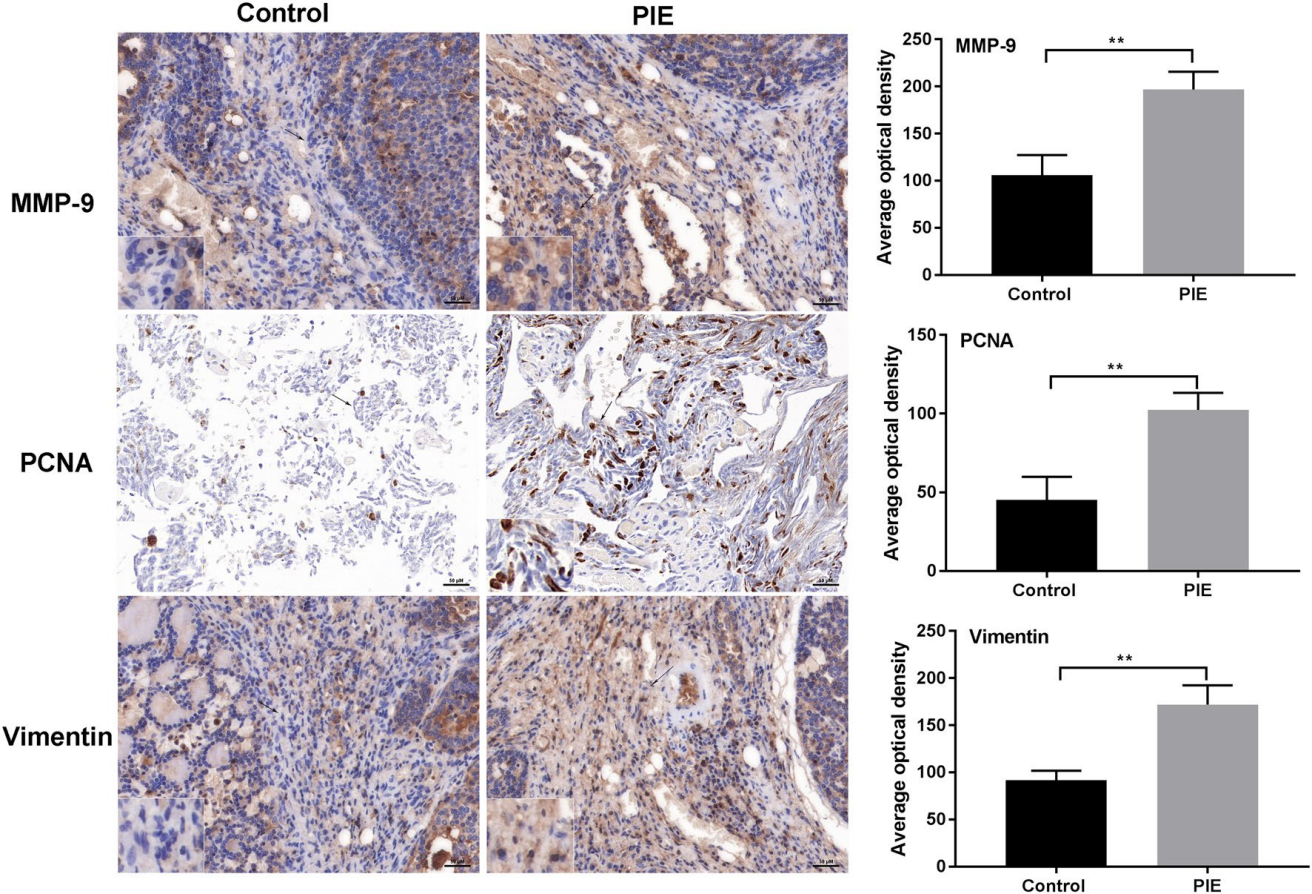
PIE-enhanced cell proliferation and EMT in female offspring mice with lung cancer. The expressions of PCNA, EMT-related proteins including MMP-9 and Vimentin in lung tissues from PIE and Control group were determined by immunohistochemistry assay, scale bar = 50 μM; **P < 0.01; PIE, prenatal inflammation exposure, EMT, epithelial-mesenchymal transition

To determine the effects of PIE on proliferation and EMT in lung cancer model, immunohistochemistry assay was performed. Results presented that the number of PCNA positive cells was higher in the PIE group than that in Control group (*P* < 0.01) (Fig. 3). Besides, EMT-markers including MMP-9 and Vimentin were up-regulated in lung tissues of PIE group (*P* < 0.01) (Fig. 3).

### PIE accelerated the offspring lung cancer progression via inducing macrophage polarization toward M2-like TAMs

**Fig. 4:**
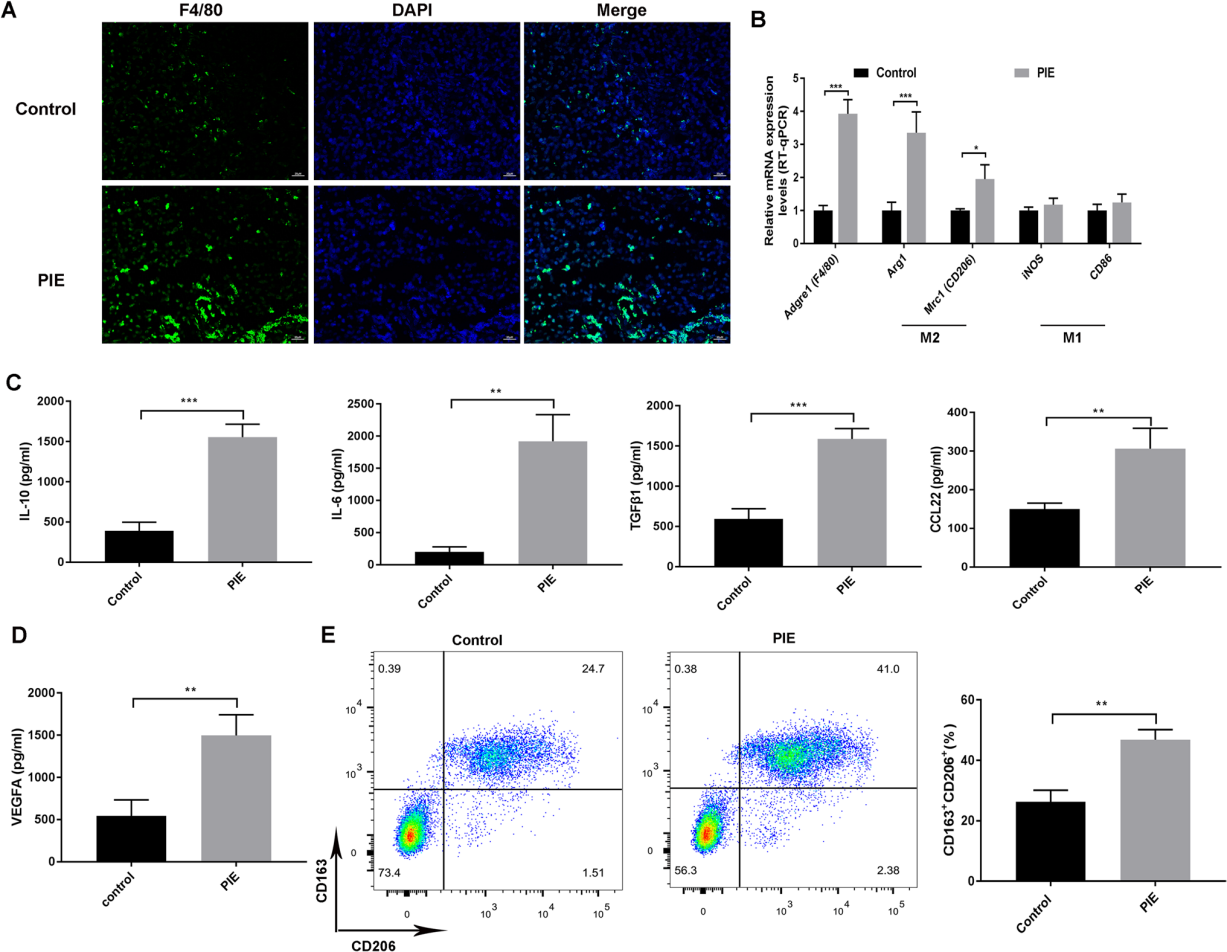
PIE-induced macrophage polarization toward M2-like TAMs in lung cancer pups. **a** Immunofluorescence staining was employed to detect the F4/80 expression in the lung tumor of offspring lung cancer model from PIE group, scale bar = 20 μM; **b** the expressions of mRNA encoding M2 macrophage markers including Arg-1, CD206 (Mrc1), and M1 macrophage markers including iNOS, and CD86 in pups of LPS-treated mothers were tested by RT-qPCR assay; **c, d** the concentration of M2 macrophages-related cytokines including IL-6, IL-10, TNF-β1 and CCL22 were detected in pups of prenatal exposed mice; **e** flow cytometry determined the PIE treatment increased the percentage of M2 macrophages markers (CD163 + CD206 +) in the sorted TAMs; ^*^P < 0.05, **P < 0.01, ***P < 0.001; PIE, prenatal inflammation exposure; TAMs, tumor-associated macrophages; data were expressed as mean ± standard deviation (SD), *n* = 6 mice in each group

It’s well known that TAMs serve the crucial function on malignant tumor initiation and progression [19]. In this study, we observed that F4/80 was remarkably more abundant in the lung tumor tissues of PIE group than that of Control group (*P* < 0.001) (Fig. 4a), indicating PIE aggravated the macrophage infiltration in offspring lung cancer model. To further verify the impacts of PIE on macrophage polarization of female mice offspring lung cancer model, we found that the expressions of mRNA encoding M2 macrophage markers including Arg-1, Mrc1 (CD206) were increased in lung cancer offspring whose prenatal mothers were exposed to LPS (*P* < 0.05), while the expressions of iNOS and CD86 (M1 macrophage markers) were not affected (*P* > 0.05) (Fig. 4b). As expected, the higher concentrations of M2 macrophages-related cytokines including IL-6, IL-10, TNF-β1 and CCL22, were also observed in pups of prenatal exposed mice (*P* < 0.01) (Fig. 4C). In line, PIE increased VEGFA expression, indicating the activation of angiogenesis at tumor locations (*P* < 0.01) (Fig. 4D). In vitro, flow cytometry data also determined the PIE treatment increased the percentage of M2 macrophages markers (CD163 + CD206 +) in the sorted TAMs (Fig. 4e). To determine whether PIE affects macrophage-induced EMT in lung cancer, lung cancer cells (A549 and SPC-A-1) were incubated with CM of TAMs from mice. Transwell assay demonstrated that A549 and SPC-A-1 treated with the CM of TAMs from PIE group exhibited increases of invasion ability (Supplementary Fig. 1a). Moreover, PIE-induced TAMs/M2 up-regulated the protein levels of N-cadherin and Vimentin, whereas down-regulated E-cadherin in A549 and SPC-A-1 (Supplementary Fig. 1b). Aforementioned findings reveled that PIE accelerated the female mice offspring lung cancer progression via M2 TAMs polarization-mediated EMT process.

### PIE activated IRE1α/XBP1 by triggering ER stress and contributed to PD-L1 expression in the TAMs of female offspring lung cancer mice

It’s well known that maternal exposure during pregnancy disrupt ER proteostasis [20] and ER stress responses is decisive for regulating TAMs function [21]. To first confirm the relationship between ER stress and M2 TAMs polarization, mouse macrophages (RAW264.7 cells) were stimulated with thapsigargin. Thapsigargin, as ER stress inducer, we first observed that ER stress-markers including GRP78/BIP, CHOP and IRE1α were higher after stimulation with thapsigargin (Supplementary Fig. 2a). Correspondingly, the expressions of mRNA encoding M2 macrophage markers including Arg-1, Mrc1 (CD206) were significantly elevated in RAW264.7 cells exposed to thapsigargin, but the expressions of M1 macrophage markers such as iNOS and CD86 did not change (Supplementary Fig. 2b).

**Fig. 5:**
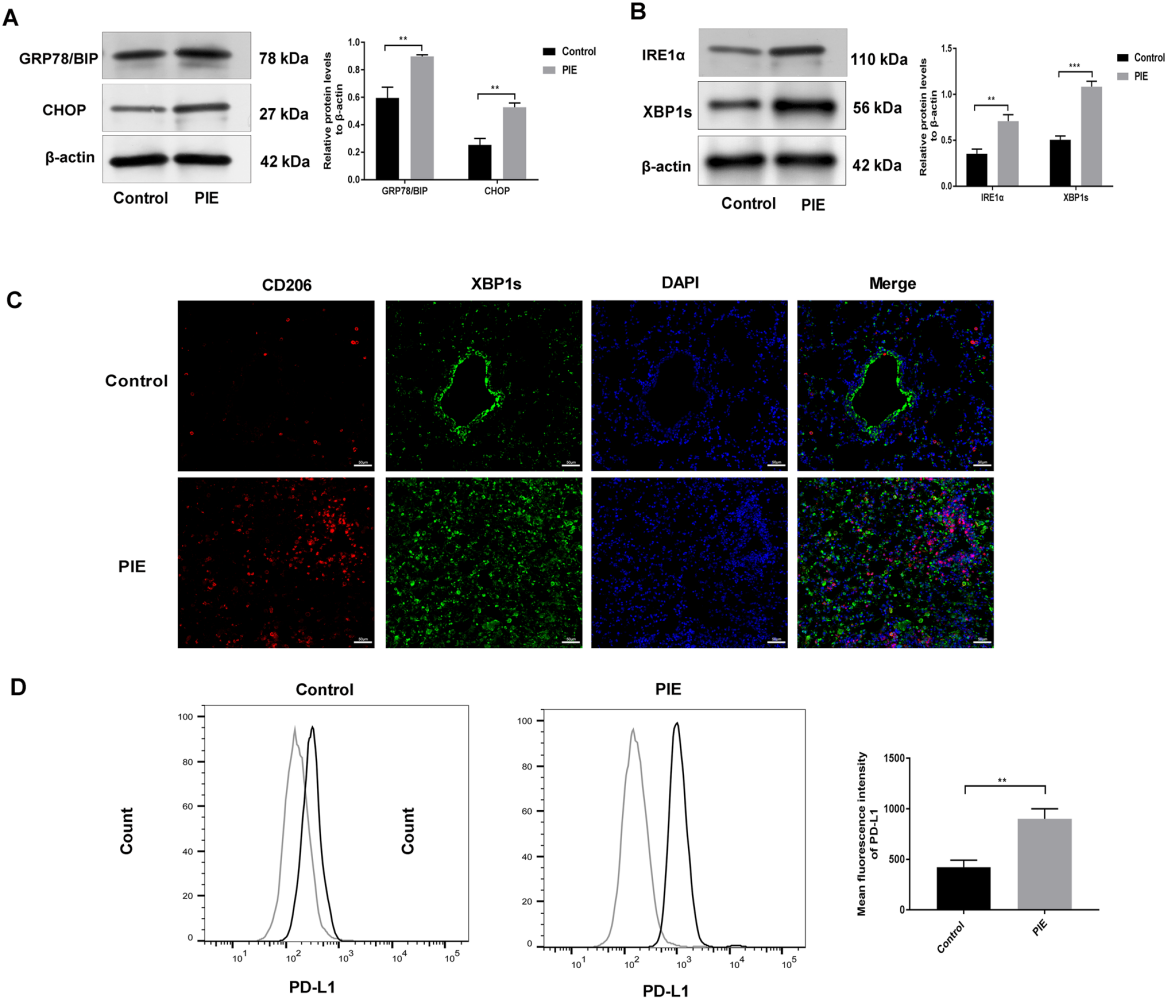
PIE activated IRE1α/XBP1 by triggering ER stress and contributed to PD-L1 expression in the TAMs of female offspring lung cancer mice. **a**, **b** Western blotting was adopted to test the ER stress-markers in macrophages derived from mice including GRP78/BIP, CHOP, IRE1α and XBP1s; **c** The positive cells of XBP1s and CD206 was determined by immunofluorescence assay, Scale bar = 50 μM; (D) The PD-L1 signal intensity in macrophages was detected by flow cytometry; **P < 0.01, ***P < 0.001; PIE, prenatal inflammation exposure; ER, endoplasmic reticulum; TAMs, tumor-associated macrophages; data were expressed as mean ± standard deviation (SD), *n* = 6 mice in each group

Consistently, in vivo results also confirmed the higher expressions of ER stress-markers including GRP78/BIP and CHOP in macrophages derived from mice of PIE group (Fig. 5a). UPR is an adaptive mechanism of ER, and its cascade activation can alleviate ER homeostasis. IRE1α/XBP1 signaling, as the most conserved process associated with UPR, we observed that the IRE1α level and its target XBP1s were obviously increased in the PIE group, by comparison with Control group (Fig. 5b). Immunofluorescence assay stated that CD206 positive cells were co-expressed with XBP1s and XBP1s expression in CD206-positive cells of PIE group was higher than that of Control group, indicating that PIE can activate XBP1 and then regulate macrophage M2 polarization (Fig. 5c). Additionally, PD-L1 signal intensity in macrophages of PIE group was higher by comparison with Control group (Fig. 5d), determining an impairment of immune response. These evidences indicated that PIE can activate XBP1 and enhance PD-L1 expression in TAMs of female offspring lung cancer mice.

### IRE1α inhibition manipulated the TAMs polarization and PD-L1 up-regulation in female offspring lung cancer mice

**Fig. 6:**
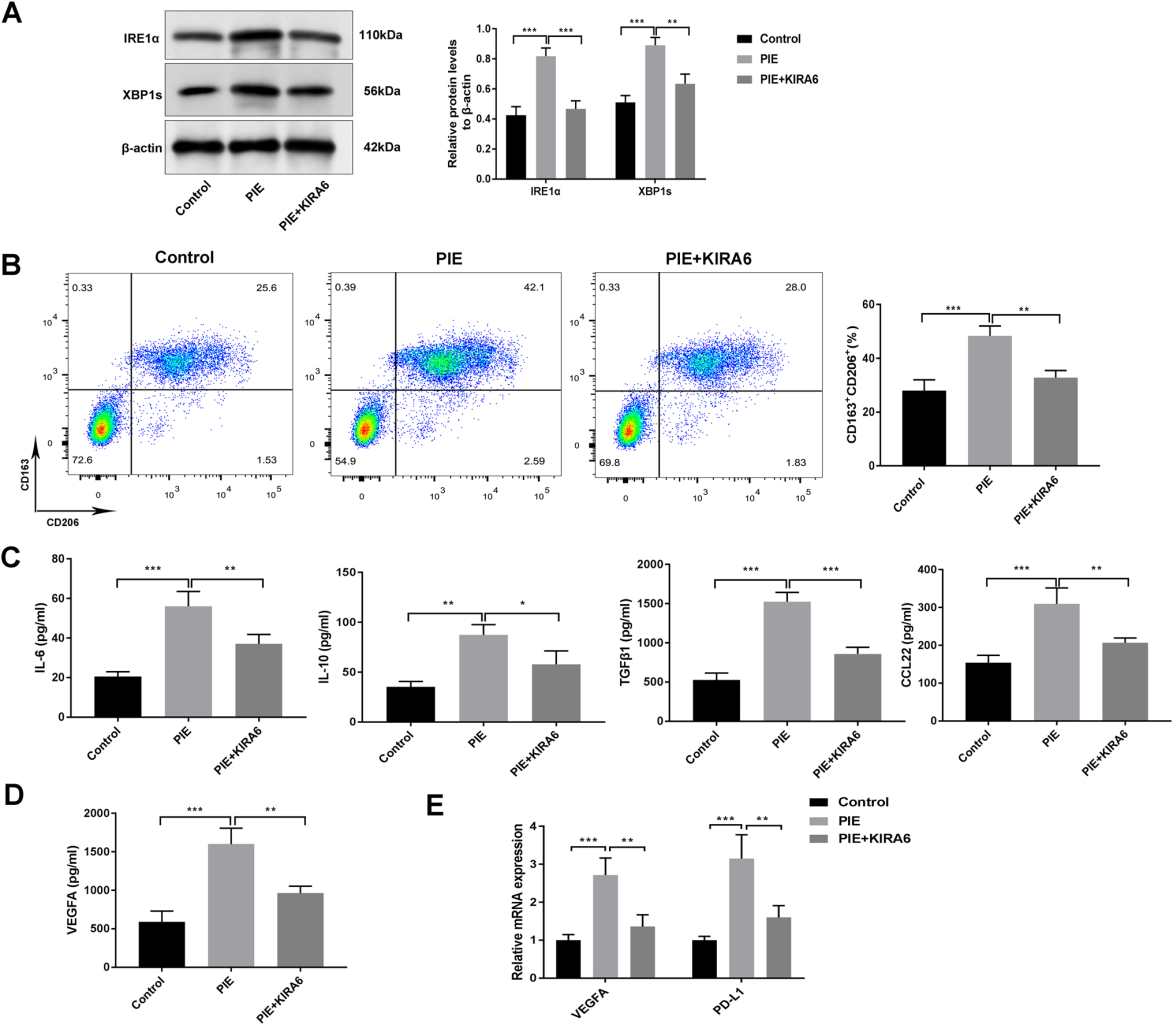
IRE1α inhibition manipulated the TAMs polarization and PD-L1 up-regulation in the female offspring lung cancer mice. **a** The levels of IRE1α and XBP1s in the macrophages derived from pups were determined by western blotting after administration with IRE1α inhibition (KIRA6); **b** the combination roles of PIE and KIRA6 on the expression of M2 macrophage-related cell surface markers (CD163 + CD206 +) were determined by flow cytometry; **c, d** the influences of KIRA6 on the levels of IL-6, IL-10, TNF-β1, CCL22 and VEGFA induced by PIE; **e** RT-qPCR was employed to test the roles of KIRA6 on the VEGFA and PD-L1 expressions induced by PIE; *P < 0.05, **P < 0.01, ***P < 0.001; PIE, prenatal inflammation exposure; TAMs, tumor-associated macrophages; data were expressed as mean ± standard deviation (SD), n = 6 mice in each group

To further verify whether UPR triggered by ER stress could influence the M2-like TAMs polarization, IRE1α inhibitor (KIRA6) was added into macrophages derived from pups. First, we observed that KIRA6 effectively decreased the levels of IRE1α and XBP1s (*P* < 0.01) (Fig. 6a). Following, flow cytometry data determined that KIRA6 effectively decreased the expression of M2 macrophage-related cell surface marker (CD163 + CD206 +) induced by PIE (*P* < 0.01) (Fig. 6b). At the same time, the levels of IL-6, IL-10, TNF-β1 and CCL22 were reversed by administration with KIRA6 (*P* < 0.05) (Fig. 6c). Furthermore, pro-angiogenic marker (VEGFA) and PD-L1 expressions in the PIE + KIRA6 group were remarkably decreased in contrast to PIE group (*P* < 0.01) (Fig. 6d, e).

The involvement of PERK pathway on TAMs polarization was also determined using GSK2656157, a PERK inhibitor (MCE, Shanghai, China). As presented in Supplementary Fig. 3, GSK2656157 efficiently inhibited the level of PERK, but had no effect on Arg1 and Mrc1 (CD206) expressions. Collectively, aforementioned results indicated that PIE-induced M2-like TAMs polarization and PD-L1 up-regulation is IRE1α/XBP1 dependent in female mouse offspring lung cancer.

### IRE1α inhibition decreased the tumorigenesis of female offspring lung cancer mice

**Fig. 7:**
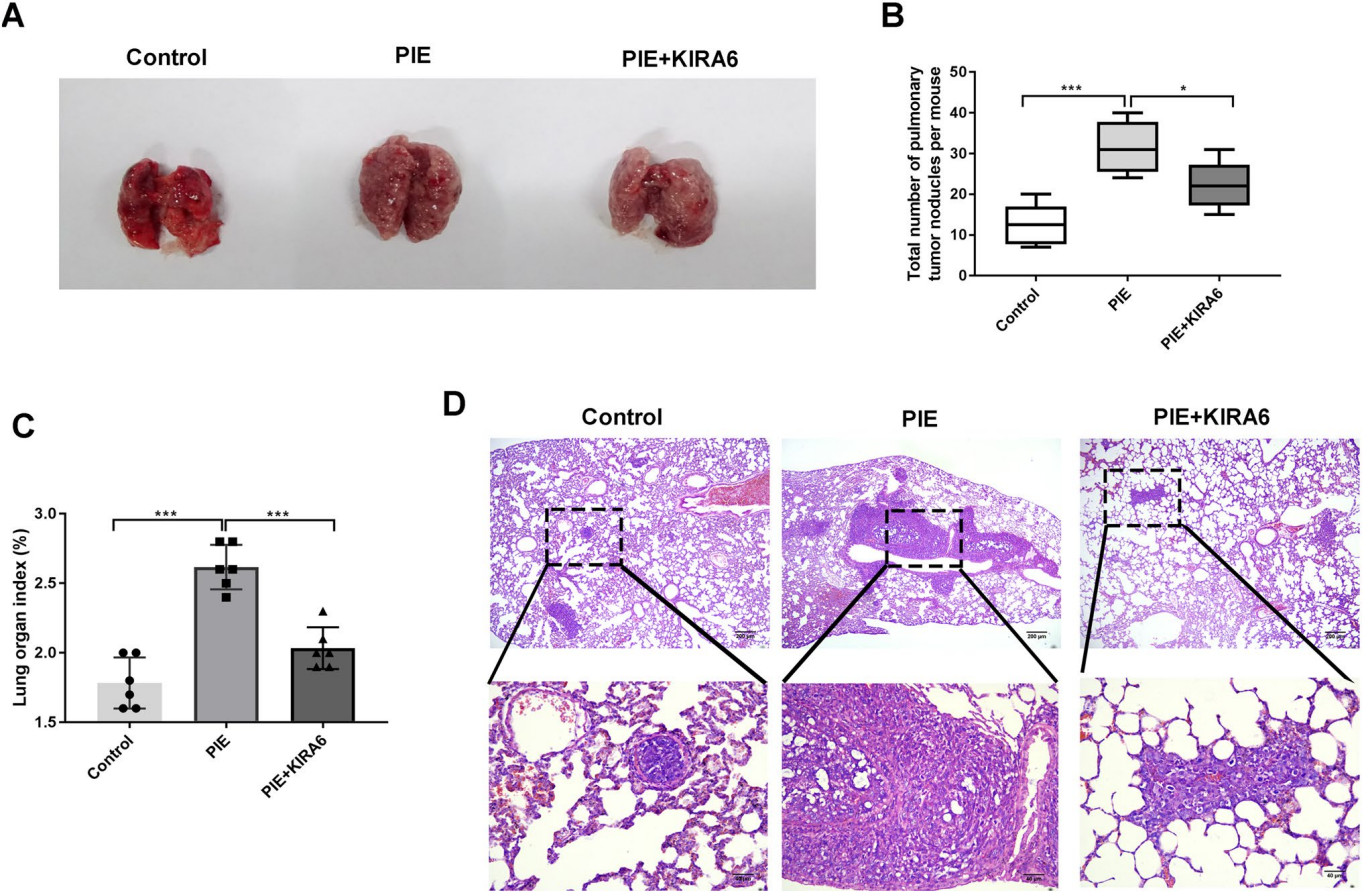
Inhibition of IRE1α decreased the metastasis of female offspring lung cancer mice. **a, b** The representative images and the number of lung surface tumor nodules were displayed; **c** lung organ index in each group was shown; **d** HE analysis revealed the roles of KIRA6 and PIE on lung lesion changes, scale bar = 200 μM or 40 μM; *P < 0.05, ***P < 0.001; PIE, prenatal inflammation exposure. Data were expressed as mean ± standard deviation (SD), *n* = 6 mice in each group

In vivo, we further investigated the influences of KIRA6 on lung tumorigenesis induced by PIE. As presented in Fig. 7a–c, the number of lung surface tumor nodules and lung organ index in PIE + KIRA6 group were less than those of PIE group (*P* < 0.05). HE analysis also revealed that lung lesion changes induced by PIE, including intrapulmonary metastases were alleviated after administration with KIRA6 (Fig. 7d). These evidences suggested that inhibition of IRE1α/UPR could be exploited to restrain the PIE-associated malignancies.

## Discussion

Prenatal exposure is known to influence the fetal development and future disease susceptibility [22]. Prior studies found that prenatal exposure can aggravate the lung dysfunction in offspring such as emphysema, asthma, and lung cancer [5, 23], however, the molecular mechanisms are poorly defined. In this study, we provided evidences that prenatal exposure to LPS was sufficient to facilitate the pro-inflammatory responses and promote tumorigenesis of lung cancer in offspring. Additionally, we also found that PIE induced macrophage M2 polarization in lung cancer pups of PIE group, which may be related to IRE1α/XBP1-mediated by ER stress.

Associations between LPS exposure in pregnancy and fetal impairment are well documented [24–26]. However, it is unclear whether exposure to LPS during pregnancy increases the risks of lung cancer in offspring. In this study, we displayed that lung surface tumor nodules and intrapulmonary metastases in PIE group were more than those of Control group. Our results were conformed to the prior study that chronic inflammation caused by prenatal exposure to smoke can notably increase the number of intrapulmonary metastases in lung cancer model of offspring [5]. Additionally, we found that the expressions of PCNA, MMP-9 and Vimentin were up-regulated in all lung tumor tissues of PIE group. A similar study has also demonstrated that the expressions of PCNA, MMP-9 and Vimentin are up-regulated in prenatal exposure mice, thereby accelerating the skin tumorigenesis [4]. PCNA, as a marker of proliferation in cancer, is highly up-regulated in lung cancer and closely relevant with the prognosis [27, 28]. Moreover, overexpression of MMP-9 and Vimentin implicates local aggressive behavior of tumors [29] and inhibition of MMP-9 and Vimentin can suppress the migratory and invasive abilities in cancer [30]. These evidences suggested that PIE may be a predisposing factor for inducing lung cancer in offspring.

TAMs, as the key components of tumor microenvironment (TME), normally express the M2-macrophage markers and corresponding cytokines after polarization. However, a few TAMs present the CD86 and CD80 markers, hence they are termed as M1-like TAMs [31]. In the current study, we found the elevations of mRNAs encoding M2 macrophage markers including Arg-1, Mrc1 (CD206), and M2 macrophages-related cytokines including IL-6, IL-10, TNF-β1, CCL22 and VEGFA in lung cancer offspring from PIE mice, while M1 macrophage markers (iNOS and CD86) were not affected, indicating that PIE induced macrophage polarization toward M2-like TAMs in lung cancer pups. Consistently, prior study has stated that inducing M2-like polarization of TAMs can maintain cancer stem cells, and then reveal tumor-promoting function [32]. Besides, our study also observed an increase of PD-L1. PD-L1, an immune checkpoint, is highly relevant with M2 TAMs [33]. Collectively, these evidences suggested that PIE can induce M2-like TAMs polarization in lung cancer pups and tumor immune escape, thereby influencing the development of lung cancer in mouse offspring.

Importantly, other mechanism by which PD-L1 expression in tumor cells has been covered to be impacted by UPR [34]. The activation of UPR exerts the vital roles on restoring the homeostasis of ER stress protein, and it also involves in the many diseases including cancer, metabolic disorders, and so on [35]. Prior studies have uncovered that ER stress responses further impact the function of infiltrating immune cells [36, 37]. IRE1α/XBP1, as the main branch of UPR signaling, can connect ER-stress and metabolic reprogramming, thereby facilitating lung cancer progression [38]. In the current study, we found the increased expressions of ER stress-markers including GRP78/BIP, CHOP, IRE1α and XBP1s in macrophages derived from mice in the PIE group, indicating that ER stress/UPR was activated by PIE in lung cancer model. The abovementioned data of our study were consistent with previous studies. For example, Cui et al. [20] have pointed that maternal exposure during pregnancy can disrupt ER proteostasis. Another study has clarified that the infection can skew the macrophage polarization toward M2-like TAMs and stimulate IRE1α/XBP1, thereby accelerating the release of pro-tumorigenic cytokine [39]. Zhao et al. [15] have also stated the pro-tumorigenic functions of XBP1 on TAMs in colon cancer. Furthermore, IRE1α/XBP1, a branch of the UPR, drives the PD-L1 up-regulation to initiate the immune dysregulation and failing immune surveillance, which have been testified [16]. In the association between IRE1α/UPR and malignancies, our data displayed that inhibition of IRE1α/UPR not only mediated the TAMs polarization and PD-L1 up-regulation, but also alleviated the metastasis of female mouse offspring lung cancer. Thus, we concluded that PIE-induced malignancies may be possible link to IRE1α/XBP1-mediated M2-like TAMs polarization and PD-L1 up-expression.

We identified that PIE can activate the inflammatory state and increase the susceptibility of offspring to lung tumorigenesis. UPR activation, especially the IRE1α/XBP1 axis, may be a key mechanism leading to M2-like TAMs polarization and up-regulation of PD-L1.

### Supplementary Information

Below is the link to the electronic supplementary material.

Supplementary Figure 1. PIE accelerated the female mouse offspring lung cancer progression via M2 TAMs polarization-mediated EMT process in lung cancer cells (A549 and SPC-A-1). (A) Transwell assay demonstrated the invasion ability of A549 and SPC-A-1 cells treated with the CM of TAMs; (B) The protein levels of N-cadherin, Vimentin and E-cadherin in A549 and SPC-A-1 cells were determined by western blotting. Data were expressed as mean ± standard deviation (SD) from three replicates, **P<0.01, *** P<0.001; CM, Conditioned medium; PIE, Prenatal inflammation exposure.
Supplementary file1 (TIF 2181 KB)

Supplementary Figure 2. The relationship between ER stress and M2 TAMs polarization in thapsigargin stimulated-macrophages (RAW264.7 cells) were confirmed. (A) The levels of ER stress-markers including GRP78/BIP, CHOP and IRE1α in the macrophages were determined by western blotting; (B) The expressions of mRNA encoding M2 macrophage markers ((Arg-1, Mrc1 (CD206)) and M1 macrophage markers (iNOS and CD86) in RAW264.7 cells were determined by RT-qPCR. Data were expressed as mean ± standard deviation (SD) from three replicates, **P<0.01, ***P<0.001; TAMs, Tumor-associated macrophages.
Supplementary file2 (TIF 396 KB)

Supplementary Figure 3. The involvement of PERK pathway on TAMs polarization was determined using GSK2656157 (a PERK inhibitor). (A) The protein levels of p-PERK was determined by western blotting; (B) The mRNA expressions of Arg1 and Mrc1 (CD206) were determined by RT-qPCR. Data were expressed as mean ± standard deviation (SD), ***P<0.001; TAMs, Tumor-associated macrophages.
Supplementary file3 (TIF 317 KB)

## Data Availability

Data URL: https://dataverse.harvard.edu/dataset.xhtml?persistentId=doi%3A10.7910%2FDVN%2FAG0A1Q&version=DRAFT.

## References

[1] Siegel RL, Miller KD, Wagle NS, Jemal A (2023). Cancer statistics, 2023. CA Cancer J Clin.

[2] Lai J, Li Q, Fu F, Zhang Y, Li Y, Liu Q, Chen H (2022). Subsolid lung adenocarcinomas: radiological, clinical and pathological features and outcomes. Semin Thorac Cardiovasc Surg.

[3] Chen P, Liu Y, Wen Y, Zhou C (2022). Non-small cell lung cancer in China. Cancer Commun (Lond).

[4] Sharma V, Gangopadhyay S, Shukla S, Chauhan A, Singh S, Singh RD, Tiwari R, Singh D, Srivastava V (2022). Prenatal exposure to arsenic promotes sterile inflammation through the Polycomb repressive element EZH2 and accelerates skin tumorigenesis in mouse. Toxicol Appl Pharmacol.

[5] Noël A, Perveen Z, Xiao R, Hammond H, Le Donne V, Legendre K, Gartia MR, Sahu S, Paulsen DB, Penn AL (2021). Mmp12 Is Upregulated by in utero second-hand smoke exposures and is a key factor contributing to aggravated lung responses in adult emphysema, asthma, and lung cancer mouse models. Front Physiol.

[6] Deng Y, Song L, Nie X, Shou W, Li X (2018). Prenatal inflammation exposure-programmed cardiovascular diseases and potential prevention. Pharmacol Ther.

[7] Ngambenjawong C, Gustafson HH, Pun SH (2017). Progress in tumor-associated macrophage (TAM)-targeted therapeutics. Adv Drug Deliv Rev.

[8] Boutilier AJ, Elsawa SF (2021). Macrophage polarization states in the tumor microenvironment. Int J Mol Sci.

[9] Larionova I, Tuguzbaeva G, Ponomaryova A, Stakheyeva M, Cherdyntseva N, Pavlov V, Choinzonov E, Kzhyshkowska J (2020). Tumor-associated macrophages in human breast, colorectal, lung, ovarian and prostate cancers. Front Oncol.

[10] Wang X, Wu Y, Gu J, Xu J (2022). Tumor-associated macrophages in lung carcinoma: from mechanism to therapy. Pathol Res Pract.

[11] Liu CH, Chen Z, Liao FT, Chung CE, Liu X, Lin YC, Keohavong P, Leikauf GD, Di YP, Chen K (2021). Lipopolysaccharide-mediated chronic inflammation promotes tobacco carcinogen-induced lung cancer and determines the efficacy of immunotherapy. Cancer Res.

[12] Jiang M, Li X, Zhang J, Lu Y, Shi Y, Zhu C, Liu Y, Qin B, Luo Z, Du Y, Luo L, Peng L, You J (2021). Dual inhibition of endoplasmic reticulum stress and oxidation stress manipulates the polarization of macrophages under hypoxia to sensitize immunotherapy. ACS Nano.

[13] Martinon F, Chen X, Lee AH, Glimcher LH (2010). TLR activation of the transcription factor XBP1 regulates innate immune responses in macrophages. Nat Immunol.

[14] Kim S, Joe Y, Kim HJ, Kim YS, Jeong SO, Pae HO, Ryter SW, Surh YJ, Chung HT (2015). Endoplasmic reticulum stress-induced IRE1α activation mediates cross-talk of GSK-3β and XBP-1 to regulate inflammatory cytokine production. J Immunol.

[15] Zhao Y, Zhang W, Huo M, Wang P, Liu X, Wang Y, Li Y, Zhou Z, Xu N, Zhu H (2021). XBP1 regulates the protumoral function of tumor-associated macrophages in human colorectal cancer. Signal Transduct Target Ther.

[16] Batista A, Rodvold JJ, Xian S, Searles SC, Lew A, Iwawaki T, Almanza G, Waller TC, Lin J, Jepsen K, Carter H, Zanetti M (2020). IRE1α regulates macrophage polarization, PD-L1 expression, and tumor survival. PLoS Biol.

[17] Petty AJ, Li A, Wang X, Dai R, Heyman B, Hsu D, Huang X, Yang Y (2019). Hedgehog signaling promotes tumor-associated macrophage polarization to suppress intratumoral CD8+ T cell recruitment. J Clin Invest.

[18] Perera F, Herbstman J (2011). Prenatal environmental exposures, epigenetics, and disease. Reprod Toxicol.

[19] Fu LQ, Du WL, Cai MH, Yao JY, Zhao YY, Mou XZ (2020). The roles of tumor-associated macrophages in tumor angiogenesis and metastasis. Cell Immunol.

[20] Cui F, Pan Q, Wang S, Zhao F, Wang R, Zhang T, Song Y, He J, Zhang H, Weng Q, Jin Y, Xia W, Li Y, Yang GY, De Vos WH, Timmermans JP, Xu S, Tang Y, Sheng X (2021). Maternal benzophenone exposure impairs hippocampus development and cognitive function in mouse offspring. Adv Sci (Weinh).

[21] Wei W, Zhang Y, Song Q, Zhang Q, Zhang X, Liu X, Wu Z, Xu X, Xu Y, Yan Y, Zhao C, Yang J (2022). Transmissible ER stress between macrophages and tumor cells configures tumor microenvironment. Cell Mol Life Sci.

[22] Ghazi T, Naidoo P, Naidoo RN, Chuturgoon AA (2021). Prenatal air pollution exposure and placental DNA methylation changes: implications on fetal development and future disease susceptibility. Cells.

[23] de Barros Mendes Lopes T, Groth EE, Veras M, Furuya TK, de Souza Xavier Costa N, Ribeiro Júnior G, Lopes FD, de Almeida FM, Cardoso WV, Saldiva PHN, Chammas R, Mauad T (2018). Pre- and postnatal exposure of mice to concentrated urban PM(2.5) decreases the number of alveoli and leads to altered lung function at an early stage of life. Environ Pollut.

[24] Guan X, Dan GR, Yang Y, Ji Y, Lai WJ, Wang FJ, Meng M, Mo BH, Huang P, You TT, Deng YF, Song L, Guo W, Yi P, Yu JH, Gao Y, Shou WN, Chen BB, Deng YC, Li XH (2022). Prenatal inflammation exposure-programmed hypertension exhibits multi-generational inheritance via disrupting DNA methylome. Acta Pharmacol Sin.

[25] Zhao J, Zhang Q, Yao D, Wang T, Ni M, Xu Y, Tang Z, Liu Z (2023). Prenatal LPS exposure promotes allergic airway inflammation via long coding RNA NONMMUT033452.2, and protein binding partner, Eef1D. Am J Respir Cell Mol Biol.

[26] Muk T, Jiang PP, Stensballe A, Skovgaard K, Sangild PT, Nguyen DN (2020). Prenatal endotoxin exposure induces fetal and neonatal renal inflammation via innate and Th1 immune activation in preterm pigs. Front Immunol.

[27] Chen X, Sun J, Wang Y (2021). Expressions of CD44, PCNA and MRP1 in lung cancer tissues and their effects on proliferation and invasion abilities of lung cancer cell line 95D. J buon.

[28] Grossi F, Loprevite M, Chiaramondia M, Ceppa P, Pera C, Ratto GB, Serrano J, Ferrara GB, Costa R, Boni L, Ardizzoni A (2003). Prognostic significance of K-ras, p53, bcl-2, PCNA, CD34 in radically resected non-small cell lung cancers. Eur J Cancer.

[29] Florescu A, Mărgăritescu C, Simionescu CE, Stepan A (2012). Immunohistochemical expression of MMP-9, TIMP-2, E-cadherin and vimentin in ameloblastomas and their implication in the local aggressive behavior of these tumors. Rom J Morphol Embryol.

[30] Baghbanzadeh A, Baghbani E, Hajiasgharzadeh K, Noorolyai S, Khaze V, Mansoori B, Shirmohamadi M, Baradaran B, Mokhtarzadeh A (2022) microRNA-193a-5p Suppresses the migratory ability of human KATO III gastric cancer cells through inhibition of vimentin and MMP-9. Adv Pharm Bull 12:169–175. 10.34172/apb.2022.018 10.34172/apb.2022.018 PMC9012914 35517889

[31] Gao J, Liang Y, Wang L (2022). Shaping polarization of tumor-associated macrophages in cancer immunotherapy. Front Immunol.

[32] Li D, Zhang Q, Li L, Chen K, Yang J, Dixit D, Gimple RC, Ci S, Lu C, Hu L, Gao J, Shan D, Li Y, Zhang J, Shi Z, Gu D, Yuan W, Wu Q, Yang K, Zhao L, Qiu Z, Lv D, Gao W, Yang H, Lin F, Wang Q, Man J, Li C, Tao W, Agnihotri S, Qian X, Shi Y, You Y, Zhang N, Rich JN, Wang X (2022). β2-microglobulin maintains glioblastoma stem cells and induces M2-like polarization of tumor-associated macrophages. Cancer Res.

[33] Sumitomo R, Hirai T, Fujita M, Murakami H, Otake Y, Huang CL (2019). PD-L1 expression on tumor-infiltrating immune cells is highly associated with M2 TAM and aggressive malignant potential in patients with resected non-small cell lung cancer. Lung Cancer.

[34] Chang LC, Chen TP, Kuo WK, Hua CC (2018). The protein expression of PDL1 is highly correlated with those of eIF2α and ATF4 in lung cancer. Dis Markers.

[35] Marciniak SJ, Chambers JE, Ron D (2022). Pharmacological targeting of endoplasmic reticulum stress in disease. Nat Rev Drug Discov.

[36] Cubillos-Ruiz JR, Bettigole SE, Glimcher LH (2017). Tumorigenic and immunosuppressive effects of endoplasmic reticulum stress in cancer. Cell.

[37] Mahadevan NR, Rodvold J, Sepulveda H, Rossi S, Drew AF, Zanetti M (2011). Transmission of endoplasmic reticulum stress and pro-inflammation from tumor cells to myeloid cells. Proc Natl Acad Sci U S A.

[38] Mao X, Yu C, Yin F, Xu W, Pan Y, Yang B, Huang T, Chen S, Luo W, Su T, Wu Z (2022). IRE1α-XBP1 regulates PDK1-dependent induction of epithelial-mesenchymal transition in non-small cell lung cancer cells. Exp Cell Res.

[39] Gilardini Montani MS, Falcinelli L, Santarelli R, Granato M, Romeo MA, Cecere N, Gonnella R, D'Orazi G, Faggioni A, Cirone M (2020). KSHV infection skews macrophage polarisation towards M2-like/TAM and activates Ire1 α-XBP1 axis up-regulating pro-tumorigenic cytokine release and PD-L1 expression. Br J Cancer.

