# Supplementary figures and images for: Prenatal inflammation exposure accelerates lung cancer tumorigenesis in offspring mouse: possible links to IRE1α/XBP1-mediated M2-like polarization of TAMs and PD-L1 up-expression
Source: Cancer Immunol Immunother. 2024 Mar 30;73(5):88. doi: 10.1007/s00262-024-03666-w (PMC10981640; doi:10.1007/s00262-024-03666-w)

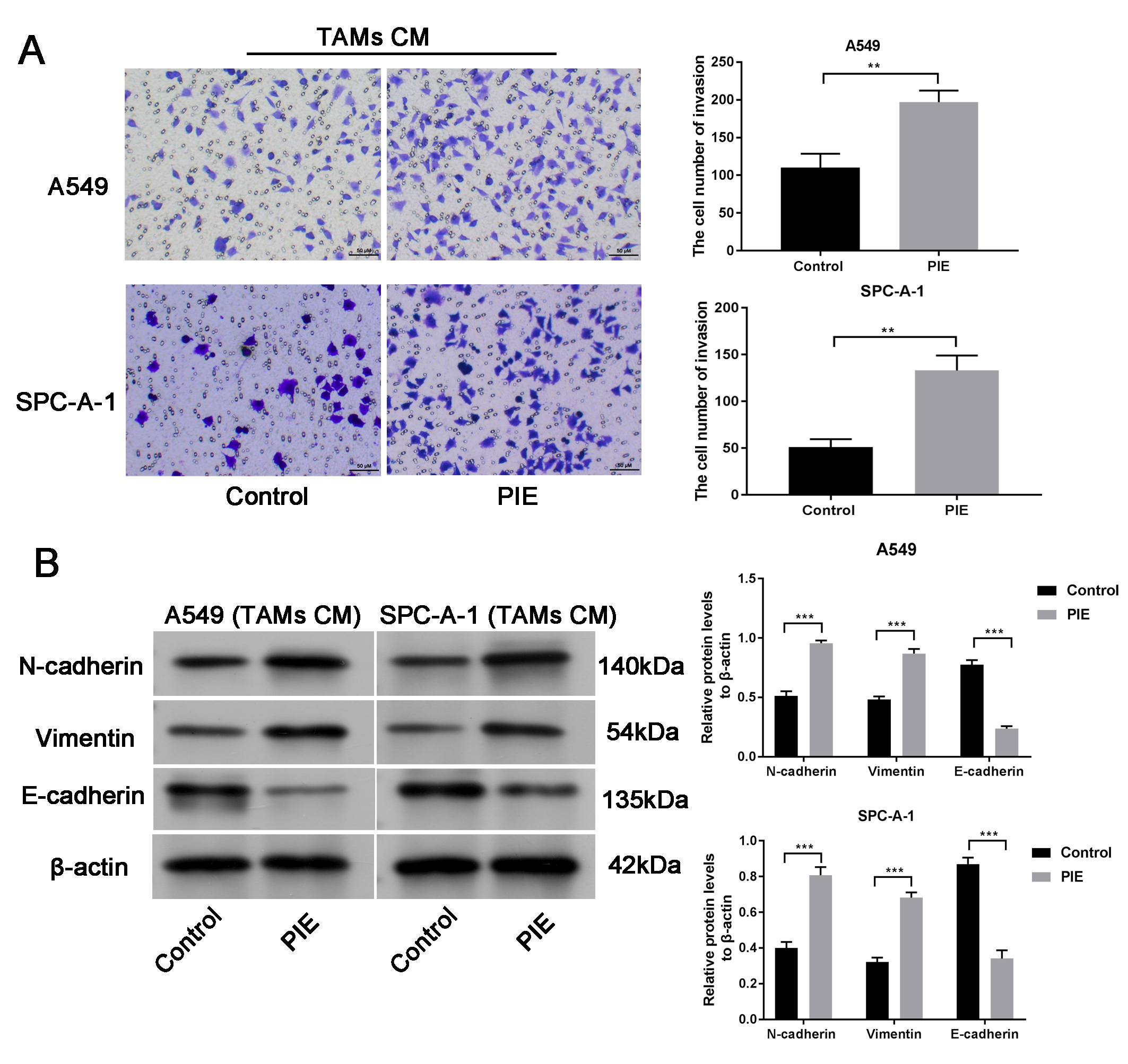

Supplement: Supplementary file 1 — Supplementary Figure 1. PIE accelerated the female mouse offspring lung cancer progression via M2 TAMs polarization-mediated EMT process in lung cancer cells (A549 and SPC-A-1). (A) Transwell assay demonstrated the invasion ability of A549 and SPC-A-1 cells treated with the CM of TAMs; (B) The protein levels of N-cadherin, Vimentin and E-cadherin in A549 and SPC-A-1 cells were determined by western blotting. Data were expressed as mean ± standard deviation (SD) from three replicates, **P<0.01, *** P<0.001; CM, Conditioned medium; PIE, Prenatal inflammation exposure. Supplementary file1 (TIF 2181 KB) [file 262_2024_3666_MOESM1_ESM.tif]

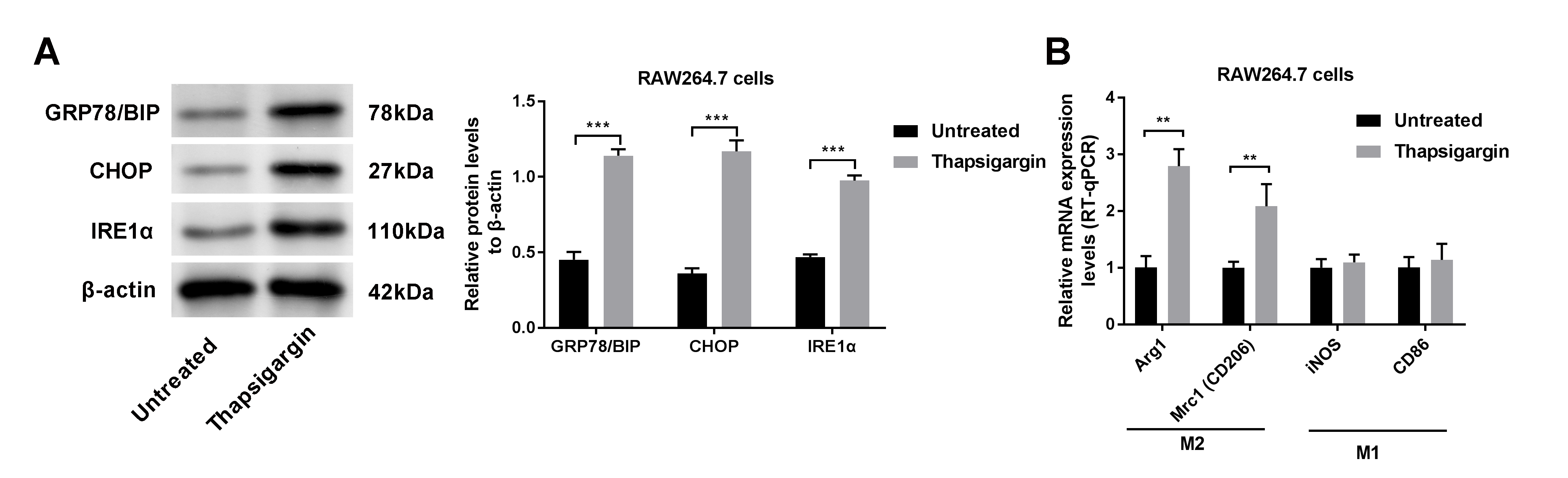

Supplement: Supplementary file 2 — Supplementary Figure 2. The relationship between ER stress and M2 TAMs polarization in thapsigargin stimulated-macrophages (RAW264.7 cells) were confirmed. (A) The levels of ER stress-markers including GRP78/BIP, CHOP and IRE1α in the macrophages were determined by western blotting; (B) The expressions of mRNA encoding M2 macrophage markers ((Arg-1, Mrc1 (CD206)) and M1 macrophage markers (iNOS and CD86) in RAW264.7 cells were determined by RT-qPCR. Data were expressed as mean ± standard deviation (SD) from three replicates, **P<0.01, ***P<0.001; TAMs, Tumor-associated macrophages. Supplementary file2 (TIF 396 KB) [file 262_2024_3666_MOESM2_ESM.tif]

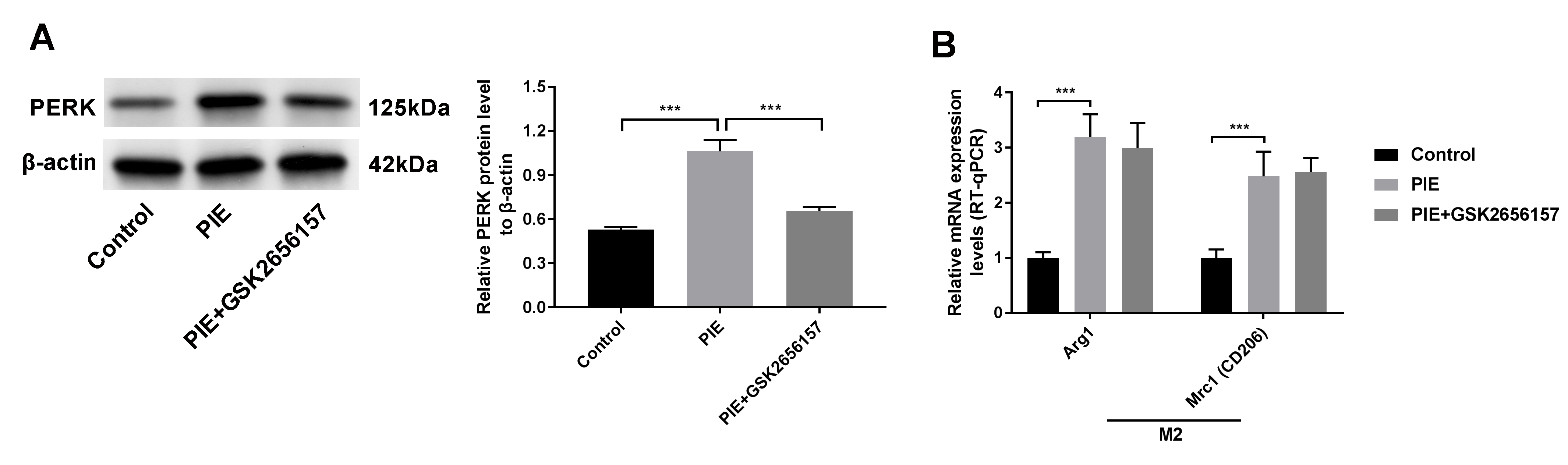

Supplement: Supplementary file 3 — Supplementary Figure 3. The involvement of PERK pathway on TAMs polarization was determined using GSK2656157 (a PERK inhibitor). (A) The protein levels of p-PERK was determined by western blotting; (B) The mRNA expressions of Arg1 and Mrc1 (CD206) were determined by RT-qPCR. Data were expressed as mean ± standard deviation (SD), ***P<0.001; TAMs, Tumor-associated macrophages. Supplementary file3 (TIF 317 KB) [file 262_2024_3666_MOESM3_ESM.tif]
